# Etio-Hematological Profile and Clinical Correlates of Outcome of Pancytopenia in Children: Experience From a Tertiary Care Center in North India

**DOI:** 10.7759/cureus.15382

**Published:** 2021-06-02

**Authors:** Muniba Alim, Nishant Verma, Archana Kumar, Vishal Pooniya, Rafey Abdul Rahman

**Affiliations:** 1 Pediatrics, King George's Medical University, Lucknow, IND; 2 Clinical Hematology, Sanjay Gandhi Postgraduate Institute of Medical Sciences, Lucknow, IND; 3 Pediatrics, Uttar Pradesh University of Medical Sciences, Etawah, IND; 4 Pediatrics, Era's Lucknow Medical College and Hospital, Lucknow, IND; 5 Pediatric Surgery, Uttar Pradesh University of Medical Sciences, Etawah, IND

**Keywords:** hematology, aplastic anemia, etiology, pancytopenia, pediatrics

## Abstract

Introduction

Pancytopenia is a clinical entity encountered in pediatric practice as a feature of various benign and malignant disorders. It describes the simultaneous presence of anemia, leucopenia and thrombocytopenia. Attempts to identify the correct etiology and gauging the severity of pancytopenia will help to determine the management and prognosis of the patients.

Objectives

To study etio-hematological profile, clinical correlates and outcome of pancytopenia in Indian children

Methods

This prospective observational study of children with pancytopenia was conducted at a tertiary care center from August 2015 to July 2016. Clinical, hematological and bone marrow studies were performed and patients were followed for one year. The collected data were statistically analyzed.

Results

Out of 84 cases, the mean age at diagnosis was 70 (70.77±4.8) months. Bone marrows showed aplastic changes in 37% and hyperplasia in 14% of patients. In our study, most common causes were aplastic anemia, acute leukemia and nutritional anemia. During the first year of follow-up, 67% pancytopenics survived and 12% succumbed (rest discontinued treatment) with ~26% of aplastic anemia (7/27 cases) and 9% of acute leukemia (2/22 cases) not surviving. Anthropometric status of patients and severity in aplastic anemia were significantly associated (p < 0.05) with outcome.

Conclusion

The data gathered support a complex picture for pancytopenia in our study population since both benign nutritional deficiencies and malignant hematological neoplasms were common. Bone marrow studies seem to be of salient use in delineating etiology. As the outcome is multifactorial, factors like anthropometry, hematological parameters have a bearing on prognosis.

## Introduction

Pancytopenia is the decrease in all three cellular elements of peripheral blood, that is, red blood cells, white blood cells and platelets leading to anemia, leukopenia and thrombocytopenia. It is encountered in clinical practice as a spectrum of multiple diseases, ranging from non-malignant conditions including drug-induced, infections or nutritional deficiencies to malignant neoplasms including primary hematological or non-hematological metastatic malignancies. Considering that both treatment and prognosis depend on the cause of pancytopenia, it is essential to reach a definitive diagnosis early [1,2]. The studies in this field have summarised that the varied causes of pancytopenia can be attributed to the geographic area, genetic differences, stringency of diagnostic criteria, and differences in the methodology used. There are varying trends in its clinical pattern, treatment modalities, and outcomes that are not only appreciated in different countries but also in different regions of a single country. From North India, there have been few studies in this area. So the present study has been undertaken to evaluate the various etiologies, clinico-hematological profile and factors responsible for poor outcome in pancytopenia in children.

## Materials and methods

Study settings

This study was conducted in a medical university, a tertiary level care center located in Uttar Pradesh, India which caters to millions of people of North India. It has a well-established department of Pediatrics which is one of the best departments in India for managing pediatric diseases including hematological disorders. The Department of Pediatrics at this University recruits children from Uttar Pradesh and adjoining states of Bihar, Uttarakhand, Madhya Pradesh, Chhattisgarh and parts of Nepal. 

Study design and data collection

This clinical observational study of children aged between one month to 15 years of age with a diagnosis of pancytopenia (hemoglobin <10 g/dL, total leukocyte count ≤4 × 10^9^/L and platelet count ≤100 × 10^9^) [3] was conducted over one year from August 2015 to July 2016. The study was conducted by the Department of Pediatrics at the lead author’s Medical University after clearance from the Institute Ethics Committee (Ref. PCM/II-B/P23 dated 29th October 2015). A written informed consent was obtained from patient’s guardians in accordance with Helsinki 's declaration. Children receiving any form of antineoplastic chemotherapy or radiotherapy were excluded from the study.

Complete clinical history of all children including present and past illness, history of drug intake, family history of hematological disease, previous blood transfusions, dietary habits and jaundice along with the findings of the clinical examination were recorded. In all patients, a complete blood count was done. As per clinical condition and feasibility, other investigations like coagulation profile, liver function tests, kidney function tests, Vit B12 levels, blood folate levels, iron studies (serum iron, ferritin and total iron-binding capacity), bone marrow smears were made to detect aplasia/hypoplasia/megaloblasts/abnormal blasts, etc., and inclusion bodies of Parvovirus B19. When needed, additional diagnostic studies like HBsAg, anti-hepatitis C virus (HCV) antibody, Parvovirus B19 Immunoglobulin M (IgM), Immunoglobulin G (IgG); Epstein-Barr virus (EBV) IgM, IgG; cytomegalovirus (CMV) IgM, IgG antibodies, anti-HIV-1 & HIV-2 antibody, gastric aspirate for acid-fast bacilli, chromosomal breakage studies were also performed.

Statistical analysis

Data were analyzed using the SPSS (Statistical Package for the Social Sciences, version 16.0, SPSS Inc, Chicago, IL, USA). Data were summarized in form of proportions and frequent tables for categorical variables. Continuous variables were summarized using means and standard deviation. Chi-square test was used for analyzing the association between various variables. A p-value of less than 0.05 was considered statistically significant.

## Results

A total of 6,208 children were admitted to the Department of Pediatrics during the study period. Out of whom, 84 children (1.4%) were diagnosed to have pancytopenia. Of these, 19 were female and 65 were male with a mean age at diagnosis of 70 months. Around 87% of children came from a rural background and the majority belonged to less affluent economic strata applying revised Kuppuswamy scales. Clinically, the majority of patients presented with complaints of fever (79%), pallor (70%) and history of previous blood transfusions (56%); and almost half of the total (49%) had episodes of bleeding like petechiae, ecchymoses and epistaxis (Table 1).

**Table 1:** Clinical characteristics of study population (n = 84).

Age in months: mean ± SD	70.77 ± 4.87
Sex	[n (%)]
Males	65 (77.4)
Female	19 (22.6)
Locality	[n (%)]
Rural	73 (86.9)
Urban	11 (13.1)
Socio-economic status	[n (%)]
Lower middle	5 (5.9)
Upper lower	47 (55.9)
Lower	32 (38.2)
Clinical features	[n (%)]
Fever	66 (78.6)
Pallor	59 (70.2)
Previous blood transfusions	47 (56)
Bleeding manifestations	41 (48.8)
Hepatomegaly	40 (47.6)
Splenomegaly	27 (32.1)
Significant lymphadenopathy	18 (21.4)
Pesticide exposure	8 (9.5)
Tremors	8 (9.5)
Jaundice	4 (4.8)
Knuckle hyperpigmentation	4 (4 .8)

In order to assess the severity, the baseline hematological parameters were further classified according to the standard age and gender-matched reference ranges (Table 2). According to statistical analysis, no significant difference was found between the major diagnostic categories (megaloblastic anemia, aplastic anemia and acute lymphoblastic leukemia [ALL]) with regards to initial hematological parameters.

**Table 2:** Distribution of hematological parameters (n = 84). Plt: platelets.

Parameters	Frequency	%
Moderate anemia	7	8.3
Severe anemia	77	91.7
No neutropenia	20	23.8
Mild neutropenia	24	28.6
Moderate neutropenia	17	20.2
Severe neutropenia	23	27.4
Lymphocytopenia present	58	69
Lymphocytopenia absent	26	31
Plt (<25,000)	39	46.4
Plt (25,000-50,000)	19	22.6
Plt (50,000-75,000)	8	9.5
Plt (75,000-100,000)	18	21.5

Bone marrow examination could be performed for definitive diagnosis of pancytopenia in 80 children and revealed aplastic changes in 37%, hyperplastic in 14% and normal cellularity in 44% of them. The bone marrow findings in various etiology (Table 3) and outcome (Table 4) of pancytopenia found in our cohort are summarized below.

**Table 3:** Bone marrow findings of study population (n = 84). ALL: acute lymphoblastic leukemia; AML: acute myeloid leukemia; HIV: human immunodeficiency virus; ITP: immune thrombocytopenia; IDA: iron-deficiency anemia; TB: tuberculosis.

	Bone marrow cellularity				Total
Diagnosis	Aplastic	Normal	Hyperplastic	Not done	
ALL	1	12	6	0	19
AML	1	2	0	0	3
Aplastic anemia	27	0	0	0	27
Complicated malaria	0	1	0	0	1
Hemolytic	0	1	1	0	2
HIV	0	2	0	0	2
Hemophagocytic syndrome	0	1	0	0	1
ITP	0	2	0	0	2
Kala azar	0	1	0	0	1
Megaloblastic anemia	0	5	4	0	9
IDA	0	0	0	2	2
TB	0	2	0	0	2
Thalassemia	0	0	1	0	1
Hypersplenism	0	1	0	0	1
Unclassified	2	7	0	2	11
Total	31	37	12	4	84

**Table 4:** Distribution of etiology and outcome in study population (n = 84). HIV: human immunodeficiency virus; ITP: immune thrombocytopenia; IDA: iron-deficiency anemia.

		Outcomes			Total
		Survived	Not survived	Discontinued treatment	
Diagnosis	Acute lymphoblastic leukemia	13	1	5	19
	Acute myeloid leukemia	2	1	0	3
	Aplastic anemia	13	7	7	27
	Complicated malaria	1	0	0	1
	Hemolytic	2	0	0	2
	Hemophagocytic syndrome	1	0	0	1
	HIV	1	1	0	2
	IDA	1	0	1	2
	ITP	1	0	1	2
	Kala azar	1	0	0	1
	Megaloblastic anemia	8	0	1	9
	Tuberculosis	2	0	0	2
	Thalassemia	1	0	0	1
	Hypersplenism	0	0	1	1
	Unclassified	9	0	2	11
Total		56	10	18	84

Patients were followed till the end of the study period and their outcome was reported in form of survival, expiry or abandonment of treatment (absconded/left treatment against medical advice/took leave, etc.). A total of 67% (56/84) pancytopenics survived, 12% (10/84) expired and the rest discontinued treatment (18/84) over one year. Aplastic anemia, acute leukemia significantly contributed to the mortality in our study (Table 4). The degree of neutropenia, lymphopenia, anemia and thrombocytopenia were not statistically associated with outcome. The severity of aplastic anemia was significantly associated with outcome; p = 0.0278 (Table 5).

**Table 5:** Association of outcome with severity of aplastic anemia (AA). p-value = 0.0278.

Severity of AA	Survived	Not survived	Discontinued treatment	Total
Non-severe	8	1	3	12
Severe	4	1	3	8
Very severe	1	5	1	7

In the under-5 population, severe malnutrition was present in 25% pts [8/32 children had mid-upper arm circumference (MUAC) less than 11.5 cm]. 38.4% (20/52) of the patient population was underweight by BMI scoring in children above five years. Anthropometric parameters, MUAC & BMI (body mass index), were significantly associated (p values of 0.0171 and 0.0045, respectively) with the outcome (Table 6).

**Table 6:** Association of outcome with anthropometrical parameters. (a) MUAC (mid-upper arm circumference), p-value = 0.0171 (chi-square test). (b) BMI (body mass index), p-value = 0.0045 (chi-square test).

Parameters: (a)MUAC, (b)BMI	Survived	Not survived	Discontinued treatment	Total
MUAC >11.5 cm	19	1	4	24
MUAC<11.5 cm	2	2	4	8
Underweight by BMI	10	5	5	20
Normal BMI	24	1	2	27
Overweight by BMI	1	1	3	5

## Discussion

In the present observational, hospital-based study, the incidence of pancytopenia was found to be 1.4% with a male to female ratio of 3.4:1 and ~85% population belonging to rural setting similar to other studies done in India [4-6]. Referral of rural population to our tertiary care center with preference to male children for health issues and less health awareness might explain the sex disparity.

We are aware that bone marrow aspiration and biopsy evaluation is of utmost importance to evaluate the causes of new-onset pancytopenia and plan further investigations [1,2,7]. In the present study, bone marrow examination was performed for 80 out of 84 patients as a pancytopenic infant of two months expired within three days and three patients refused for bone marrow. Out of 31 bone marrows that were found to be acellular/aplastic, two patients were labelled as transient cytopenia (probably viral in origin; as myelosuppression is accompanied by EBV and CMV positivity) wherein the counts recovered after seven to 10 days. The patients were asked to remain in follow-up. Here, despite an aplastic marrow a firm diagnosis of aplastic anemia couldn’t be made. As substantiated by Weinzierl who emphasized that a significant fraction of cases in both children and adults demonstrated nonspecific marrow findings that required clinical follow-up and/or repeat biopsy for definitive diagnosis in a detailed review published a few years back [1].

Data obtained for relative frequency of different causes of pancytopenia was compared with other studies in and around India. Aplastic anemia was the most common cause accounting for 27 cases (32.1%) in this study. Comparing with other studies, a similar incidence to our study findings was demonstrated by Naseem et al [8]. Savage et al [9] also reported it to be the second most common cause (26.1%). Kumar et al [3] found it to be the commonest cause (29.5%) of pancytopenia among adults at a hematology center, due to the high proportion of referred cases at their center. As a hematological disorder with severe dyscrasias, aplastic anemia is more prevalent in Asia compared to the West. European studies indicate the annual incidence of only two new cases per million of population [10]. However, studies done in China [11] and Thailand [12] reported that the frequency of aplastic anemia is three folds greater than that of the western countries of the world. The age of aplastic anemia patients varied from two to 14 years with a mean age of 8.33 years in the present study which is similar to the results of Khodke et al [4]. Sex distribution of aplastic anemia in our study was found to be 3.5:1 (males: females) as opposed to ~1:1 of Turkey [13] and 1.06 in a French study [14]. This male preponderance in the demographic profile is due to preferential reporting of the disease from the male counterparts of the society, especially from the rural areas, which constitutes 77.7% of the study group of aplastic anemia. No etiologic factor could be implicated in the majority of the cases in the present study with aplastic anemia similar to findings by Mishra et al [15].

The second important cause in our study was leukemia diagnosed in 26.2% (22/84) patients comparable to 17.4 % incidence as reported by Rathod et al [16]. Amidst our 22 patients, only three had acute myeloid leukemia while the rest 19 belonged to acute lymphoblastic leukemia; the most common type of childhood malignancy. In the present study, mean age of leukemia patients was found to be 5.5 years (range 2-11 years). Morphological breakup of leukemia population reveals normocytic picture in 10 (nine with increased red cell distribution width [RDW] whereas in one RDW was not sent initially) microcytic in eight and macrocytic in four patients. The picture may have been confounded by haematinics, prior transfusions and iron, folate & Vit B12 deficiency, etc.

The third most common cause was found to be nutritional anemia with an incidence of 13.1% comparable to the findings of Naseem et al [8] and Kumar et al [17]. Another interesting observation in the present study was that vitamin B12 deficiency was found to be more common than folate deficiency in patients with megaloblastic anemia -consistent with the results of similar studies conducted in India and around [18,19]. Vit B12 levels and folate levels could be done in 46 patients only due to affordability issues and 16 of them (35%) had deficient B12 levels- present in seven patients with nutritional anemia, five patients of acute leukemia, two aplastic anemia, one with transient cytopenia and one with miliary TB. Folate deficiency was reported in 3/46 patients - one with thalassemia, miliary TB and a patient with leukemia. The miscellaneous group comprised of infections and hematological disorders like malaria, TB, hemophagocytic lymphohistiocytosis (HLH), thalassemia, unclassified, etc. Results came positive for Parvovirus B19 in 8%, EBV in 7% CMV in 1%, Hepatitis in 2.4%, gastric aspirate found acid-fast bacilli in ~4% and paroxysmal nocturnal hemoglobinuria (PNH) clones were detected in ~5% cases. Most patients in this group had counts recovery within 10 days; probably transient (secondary to viral; could not be assayed). So, it is implied that even benign causes might present with severe pancytopenia if symptoms are neglected for long or diagnosis is delayed.

This observational study also aimed to study factors associated with poor outcome in pancytopenic patients. Patient's outcome depends on the etiology of pancytopenia, treatment modalities, supportive care, and the era in which results are analyzed. This is exemplified by advancements in the management of aplastic anemia over the decades. In a large series conducted before 1957, Wolff [20] found a survival rate of only 3% in aplastic anemia whereas in a 2004 series [21], 12% had a spontaneous resolution, 21% remained in the moderate range and 67% progressed to severe aplastic anemia. In our study, the mortality was found to be highest in the age group of five to 10 years in which aplastic anemia and acute leukemia were the causative factors. Seven out of 10 expired children were boys but should not give a false impression of more vulnerability in males as there is no equal representation of both sexes in the study population (male:female = 3.4:1 in our study population). Notably, the proportion of deaths in males is 11% (7/65) which is less than that in females at 15.7% (3/19). Socio-cultural perspective of preferential presentation for treatment in male children and tendency for discontinuing treatment in female children by guardians especially hesitation and reluctance for arranging blood for advised transfusions can explain the reported data.

The etiology-wise morbidity and mortality distribution highlights some interesting observations (Table 3). The highest contributor to mortality in our data series was made by aplastic anemia, it was seen that despite the same protocol of immunosuppressive therapy (ATG along with cyclosporine) and hospital-based transfusions to all the 27 aplastic anemia pts in our study, outcome differed across groups. This was seen to depend on the severity of aplastic anemia (p-value =0.0278; Table 5), sepsis control along with prophylactic and therapeutic blood transfusions. The patients with very severe aplastic anemia had the worst outcome with only one of the seven patients surviving despite upgraded antibiotics, blood transfusions (Table 5). The confounding reasons could have been poor pre-morbid state, severe infections considering the low blood counts and more need for transfusions before admission. Kulkarni (2015) et al [22] asserted that the clinical presentation of patients with pancytopenia clearly diﬀered from the ALL patients without pancytopenia at presentation. These clinical indicators may denote less aggressive nature of the ALL blasts and of the disease in these patients and may potentially point to a distinct biology of disease in them [22]. This was also seen in our study with only two out of 22 leukemia patients not surviving (one expired after the beginning of induction chemotherapy). Hence, 70% of the total deaths occurred in patients with aplastic anemia, 20% in leukemia and benign diseases like megaloblastic anemia, hemolytic anemia and infections like kala azar, etc., did not add to morbidity.

Patients younger than five years old with severe wasting and smaller MUAC had a poor outcome (Table 6) so did underweight pancytopenic children above five years; contributed by etiology, pre-morbid state and infections. We found that the negative outcome of children is significantly associated with low anthropometrical values (p-value < 0.05). All these factors were related to nutritional status of child and finally to overall immunity and capability of him/her to cope up with infections, transfusion-related complications and severe stage of anemia. Though not found to be statistically significant (p-value > 0.05) but severe anemia was observed in all the 10 expired patients. Also lymphocytopenia (seen against standard age-specific reference ranges) was present in 70% of the expired patients. Similarly, 40% of the expired patients had severe neutropenia. Obviously, these isolated findings should not be extrapolated as the outcome is an amalgamation of multiple factors.

The present study adds to socio-demographic and clinical profile of the varied spectrum of etiology and severity of pancytopenia in children, of which potentially treatable and reversible miscellaneous causes like infections and nutritional deficiencies emerged as a causative factor in a notable percentage of the pancytopenia cases. However, the majority was accounted for by the acute leukemia and aplastic anemia at our referral tertiary care center. Detailed hematological profiles of the entities were traced but failed to show any marked difference at initial presentation. Emerging use of estimation of serum vitamin B12, folate levels, viral markers, PNH clones and parasitological tests along with the use of invasive procedures like bone marrow aspiration aid in evaluating cytopenia and in its definitive management. Low counts increase morbidity and recovery rates become poor getting further clouded by the need for recurrent transfusions as well as active sepsis control. Notable socio-cultural perspective in a developing country like India in form of socio-demographic variables like gender, locality (urban vs rural) tend to have a significant effect as they play a role in a timely presentation to health service, causative exposure (to pesticides), replacement blood donation and general health condition. Cultural factors like denial for replacement blood transfusion [23], nutritional anemia and worm infestations might skew the expected clinical findings as seen in the present study.

The present study has limitations in form of limited patient population (84), follow-up period (one year) and logistic issues (unaffordability for some investigations). As already stated, the study setting was in a tertiary care center located in the capital of a densely populated state getting referrals from north India, so skewness in form of a higher incidence of malignant causes may be expected. A more detailed study with a greater duration of follow-up may outline the course of disease and effects of each of these variables on final outcome.

## Conclusions

The data gathered support a complex picture for pancytopenia in our study population since both benign nutritional deficiencies and malignant hematological neoplasms were noted. A good clinical correlation is of utmost importance to evaluate each specific case and plan for further hematological evaluation and appropriate management as the outcome is multifactorial.
